# Production of protein-rich fungal biomass from pistachio dehulling waste using edible *Neurospora intermedia*

**DOI:** 10.1038/s41598-024-81941-7

**Published:** 2025-02-18

**Authors:** Javad Toghiani, Narges Fallah, Bahram Nasernejad, Amir Mahboubi, Mohammad J. Taherzadeh, Neda Afsham

**Affiliations:** 1 Department of Chemical Engineering, Amirkabir University of Technology, Tehran, 15875-4413 Iran; 2 Swedish Centre for Resource Recovery, University of Borås, 501 90 Borås, Sweden

**Keywords:** Filamentous fungi, *Neurospora Intermedia*, Pistachio dehulling waste, Protein-rich biomass, Waste valorization, Biotechnology, Environmental biotechnology

## Abstract

Pistachio dehulling waste, known as Pistachio byproduct mixture (PBM), is a valuable resource that is often overlooked. An effective sustainable approach involves utilizing this agricultural waste through a fermentation process using edible filamentous fungi, demonstrating potential applications in nutrition and animal feed. The focus of this study was on converting PBM extract obtained from a hot water extraction pre-treatment into a protein-rich fungal biomass of *Neurospora intermedia*. The optimal conditions for growth were achieved at 72 h, pH 5.5, and 30 °C which are achieved by one-factor-at-a-time approach (OFAT), resulting in 6.7 g/L of dried fungal biomass, with a protein content of 20.4%. The conversion efficiency, expressed as grams of fungal biomass per gram of initial Total COD, was 0.37 g/g, highlighting the significant potential of PBM extract with high COD levels and low sugar content for fermentation processes. Additionally, an investigation was carried out to assess the impact of inoculation method, culture adaptation, COD/N ratio, and pH control on fungal biomass growth during cultivation. The results of optimal conditions with response of fungal biomass growth showed production of 0.44, 0.45, and 0.49 g of fungal biomass per gram of initial total COD, with protein contents of 20.2%, 27.1%, and 18.6%, respectively, leading to improved fungal biomass yield. The resulting protein-rich fungal biomass with a focus on the biorefinery platform to complete the value-added cycle, holds promise for applications in various sectors including food, animal feed, biochemical, and biomaterial industries.

## Introduction

Pistachio (*Pistacia vera L*.) is a tree nut extensively cultivated in various regions globally, including Iran and the United States, owing to its manifold nutritional and health advantages. The escalation in pistachio demand has led to a substantial upsurge in production, consequently resulting in an increase in the volume of waste produced during the processing phase. The waste from pistachio dehulling comprises leaves, clusters, and green hulls, collectively referred to as pistachio byproduct mixture (PBM). PBM is annually generated in significant amounts (approximately 600,000 metric tons in 2023) and is rich in nutrients like phenolic compounds (approximately 8.6–9.3% on a dry basis), carbohydrates (NDF of 25.5–31.8%), and minerals^1,2^.

Owing to the obstinate composition of PBM as a lignocellulosic biomass characterized by limited enzyme and microorganism accessibility to its internal structure, pretreatment becomes imperative prior to engaging in bioconversion procedures. Moreover, inadequate handling of PBM, such as its disposal in agricultural areas and utilization as animal fodder without pretreatment, has led to the emergence of numerous environmental and economic challenges. Various strategies have been postulated to transform this refuse into valuable commodities utilizing a biorefinery approach, which advocates for sustainable biomass processing aimed at generating bioenergy, biofuels, and bio-based goods^3–5^. Physical methods like sieving and grinding are viable for transforming PBM into smaller particles suitable for soil enhancement. Additional physical techniques, such as extraction, along with certain chemical or physicochemical methods like the microwave-assisted approach, can be utilized for the extraction of bioactive compounds such as polyphenols and flavonoids from PBM. These compounds exhibit promising potential for applications in the food and pharmaceutical sectors^6–8^. Moreover, Thermochemical techniques, like pyrolysis, have the potential to generate activated carbon or bio-oil. Nevertheless, these approaches frequently necessitate severe operational parameters, aggressive chemical agents, and result in the production of dangerous waste byproducts^2^.

Most existing studies on pistachio byproduct valorization have primarily concentrated on the extraction of compounds from the waste material, with limited emphasis placed on biological valorization strategies. The utilization of biological approaches, including fermentation and anaerobic digestion, presents more eco-friendly and sustainable solutions for valorizing pistachio byproducts, characterized by reduced energy demands^9,10^. Among these methodologies, the employment of filamentous fungi as a robust enzymatic facility is regarded as a promising approach for the transformation of agro-industrial residues like wheat straw, rice hull, apple pomace, and thin stillage into beneficial commodities such as metabolites (e.g. citric acid, gluconic acid, ethanol, pigment, etc.), biomass (e.g. feed, chitin, fatty acid, lipids, etc.), and enzymes^11^. *Neurospora intermedia*, an ascomycete fungus, is considered one of the filamentous fungi that has undergone extensive examination in the context of valorizing agricultural waste. The enzymes that are synthesized by *N. intermedia* possess the capability to degrade intricate starch and cellulose configurations into more elementary sugars, which in turn can be utilized in the generation of bioproducts enriched with protein, fat, and essential amino acids. Additionally, it has been documented that this particular fungus exhibits the capacity to generate a variety of supplementary metabolites of value, such as ethanol and organic acids, derived from lignocellulosic biomass and agro-industrial waste^12–14^ .

*N*,* intermedia* can be grown using submerge and solid-state fermentation (SSF). Although SSF is more energy efficient, the process parameters control and downstream processes are more difficult^15^. In the context of submerged fermentation, the collection of the fungal biomass generated for subsequent utilization holds greater relevance. The cultivation of *N. intermedia* hinges largely on pivotal parameters such as temperature, medium pH, agitation, time, and inoculum. Through the strategic fine-tuning of these variables, and aligning them with the specific agro-waste utilized and the method of pretreatment, one can feasibly generate a protein-rich biomass boasting a substantial yield. The optimal conditions conducive to *N. intermedia* growth typically encompass a temperature range of 30℃, a pH range of 5.5–6.5, an agitation level of 100–200 rpm, a duration of 3–5 days, and an inoculum quantity ranging from 1 to 5% of the medium volume^16,17^.

In a study, Gmoser, et al.^12^ converted waste stream from bioethanol industry (thin stillage) and bread waste into protein and pigment using *N. intermedia*, combining submerged and SSF. In the optimum conditions, 81 kg ethanol and 65 kg fungal biomass per ton dry weight of thin stillage was produced in the submerge process. In addition, the protein content in waste bread increased by 161% and 1.2 kg pigment per ton of waste bread was obtained in SSF as the second fermentation step. Another investigation examined the transformation of expired yogurt, cheese whey, sour milk, and cream (dairy waste) into premium quality biomass suitable for either feed or food purposes, as well as the production of biobased chemicals like ethanol and glycerol. The cultivation of *A. oryzae* and *N. intermedia* under optimal conditions yielded approximately 0.12–0.47 g of biomass per gram of waste (7–16 g/L)^18^.

Pretreatment of Pistachio dehulling waste, a type of lignocellulosic waste, is essential to facilitate fermentation processes. The primary approaches for this pretreatment involve hydrothermal and chemical methods. One effective method is the application of hot water treatment to degrade the waste structure and extract its components for submerged fermentation. This method offers various advantages, such as cost-effectiveness, low toxicity, and minimal energy requirements. Moreover, it is environmentally friendly as it does not generate any harmful byproducts^18–20^.

To the best of our knowledge, despite the abundant availability of PBM and its high potential for bioconversion, there is a significant gap in the literature regarding the application of biological approaches such as fermentation to valorize this waste stream. While a few studies have explored the utilization of pistachio dehulling waste, these have primarily focused on its use as animal feed or compost. In contrast, our research pioneers the use of edible filamentous fungi to convert PBM into a valuable protein-rich fungal biomass. The study focuses on optimizing operational parameters like cultivation duration, initial pH levels, and temperature for submerged cultivation of *N. intermedia* by one-factor at a time approach (OFAT) to enhance biomass production. Furthermore, the investigation extends to studying the impact of different inoculation methods, pH adjustments during cultivation, and nitrogen source supplementation on fungal growth.

## Material and method

### Substrate

Pistachio dehulling waste include leaves, clusters and green hulls also known as pistachio byproduct mixture (PBM), were obtained from pistachio processing terminal and airdried at ambient temperature. In order to perform the relevant analyses, the dried wastes were milled and screened with a mesh size of 35–70 and prepared for further tests. All other chemicals (glucose, agar, yeast extract, NaNO_3_, NaOH and H_2_SO_4_) added to the cultivation medium were of analytical grade and purchased from MERCK.

### Filamentous fungi

*Neurospora intermedia* as an ascomycete edible filamentous fungus was provided from the Industrial Microorganism Collection Center of Iran (PTCC No. of 5291). The fungus was maintained on medium plates containing 20 g/ L glucose, 15 g/ L agar and 4 g/ L Yeast extract; the medium was autoclaved at 121 °C for 20 min. A pre-grown fungal plate was flooded with 20 mL of sterile ultrapure water and a L-shaped disposable plastic spreader was used to bring the spores into suspension. Then, 0.1 mL of the spore suspension was inoculated and spread into new plates. The inoculated plates were incubated at 30 °C for 3 days followed by storage at 4 °C until use.

### Pretreatment

The pretreatment of hot water was conducted under optimized operational parameters, including a temperature of 90 °C and a duration of 90 min, with a substrate-to-solution ratio of 0.1 in sealed glass flasks of 0.5 L to prevent water loss through evaporation. The reactor was positioned in a water bath within the specified range throughout the reaction period. Subsequently, upon completion of the pretreatments, the samples underwent filtration using filter paper with an 8 μm pore size. The resulting liquid was utilized as the primary culture medium in the subsequent stages. Furthermore, to explore the impact of the initial concentration of PBM and its inhibition, multiple trials were carried out using concentrations of 5, 10, 15, and 20 g PBM/100 g solution. Owing to the high water absorption capacity of PBM and the low PBM extraction following pretreatment with elevated PBM concentrations, a consistent PBM-to-solution ratio of 0.1 (w/w) was maintained in all pretreatment processes for cultivating filamentous fungi.

### Fungi cultivations

Cultivation procedures were executed in 250 mL cotton-plugged Erlenmeyer shake flasks containing 100 mL of medium. Fungal cultivations were performed at initial pH levels of 3, 4.5, 5.5, and 7 (adjusted using 0.1 mol/L H_2_SO_4_ and 0.5 mol/L NaOH), various cultivation durations (6, 12, 24, 36, 48, 60, 72, 96, and 120 h), and temperatures ranging from 20 to 45 °C. All cultivation mediums underwent autoclaving at 121 °C for 20 min. The initial inoculation comprised 2.5% v/v of spore suspension. All experiments were conducted in a shaker incubator at 120 rpm. Following the completion of fungal growth, measurements of pH and medium volume were taken. Subsequently, the fungal biomass produced was harvested using a sieve with a pore size of 1 mm2 and rinsed thrice with ultrapure water. Liquid samples were collected periodically and stored at -20 °C for subsequent analysis via HPLC and GC-MS. The harvested biomass was dried for 24 h in an oven at 60 °C, and the biomass yields were quantified as grams of biomass per liter of PBM extract. All experiments were replicated, and the outcomes were depicted in figures and tables, presented as mean values with a double standard deviation.

Following the optimization of operational parameters, an examination of the inoculation methods’ effects involved adding spores to the diluted primary culture media in one trial. After 24 h, 10% v/v of the pre-cultured solution was introduced into the primary culture. Additionally, pH adjustments during cultivation and investigating the influence of adding a nitrogen source (NaNO3) on fungal growth were also carried out.

### Analytical methods

Total protein content in dry biomass was measured by analyzing nitrogen content using the Kjeldahl method according to Mahboubi, et al.^21^. The crude protein content was calculated by applying a nitrogen-to-protein conversion factor of 6.25. For volatile solids (VS) and ash analysis, 0.2 g of fungal biomass and PBM in pre-dried crucibles were ignited in a muffle furnace for 4 h at 550 °C ± 25 °C. The amount of ash was measured using crucible weight difference. Sugars and phenolic compounds were analyzed using HPLC (high-performance liquid chromatography) equipped with Nucleosil 100-NH_2_ (Mobile phase: Acetonitrile: water of 90:10 v/v) with a flow rate of 1 mL/min and operated at 30 °C and Ultisil XB-C18 column eluted with buffer phosphate (pH 2.7) as eluent A and methanol as eluent B with a flow rate of 1 mL/min and operated at 30 °C, respectively. The chemical oxygen demand (COD) of the PBM extract was measured by closed reflux method based on the standard method^22^. PBM extract which obtained after pretreatment was characterized using GC-MS (The Agilent 6890 N GC with 5973 N MS, Rxi 5ms column). The morphology of the fungal biomass was determined by visual examination of submerged culture (mycelium or pellets).

## Result and discussion

The proliferation of edible ascomycete *N. intermedia* in the presence of PBM extract, obtained through hot water pretreatment, as a substrate was examined. Successful cultivation of the fungus was achieved in PBM extract as the growth medium. Various factors such as cultivation time, initial pH, temperature, nitrogen source addition, cultivation strategy, and pH regulation during fungal growth were extensively explored.

### Pretreatment and medium preparation

The characteristics of PBM were analyzed before conducting pretreatment experiments, as delineated in Table 1.

**Table 1:** The PBM characteristics.

Parameter	Average quantity
Protein	9.9%
Phosphorus	0.06%
Moisture	4.5%
Total Solid (TS)	95.2%
VS	89.8% of TS
Ash	10.2% of TS
Total extract with water (after 24 h)	48% of received sample
Total carbohydrate in aqueous phase after extraction with water (after 24 h)	0.62 g/L
Neutral Detergent Fiber (NDF)	39.4%
Acid Detergent Fiber (ADF)	24.5%
Acid Detergent Lignin (ADL)	12.3%
Gallic acid	1650^1^ and 1795^2^ ppm
3,4-Dihydroxy benzoic acid	792^1^ and 972^2^ ppm
Rutin	530^1^ and 650^2^ ppm
Quercetin	60^1^ and67^2^ ppm

1. Ethanol extraction of PBM.

2. Methanol extraction of PBM.

The analysis revealed that the total carbohydrate content in the aqueous phase is negligible compared to the overall extract (Table 1), hence insufficient for substantial growth of filamentous fungi. In contrast to carbohydrate levels, phenolic compounds and the total extract quantity, or in simpler terms, the COD amount, exhibit promising potential for fostering the growth of *N. intermedia.*

In the context of hot water extraction pretreatment, the resultant liquid serves as the primary culture medium. During this pretreatment process, approximately 38% by weight of PBM (water-soluble extract providing carbon and nutrient sources for the culture medium) is transferred to the aqueous phase. The total COD of the medium was determined to be 17.9 g/L. The Kjeldahl Nitrogen content of the medium was inconsequential (about 0.02% by weight). Furthermore, the HPLC analysis quantified the sugar and phenolic compound levels in the culture medium. The measured amounts of glucose and rhamnose were 0.28 and 0.52 g/L, respectively, corroborating the earlier findings regarding the insignificance of sugar in the PBM culture medium. Additionally, the quantities of gallic acid and quercetin as phenolic compounds were identified as 2936 and 67 ppm, respectively.

Subsequent GC-MS analysis of the medium led to the identification of 72 compounds. Notably, Acetaldehyde (36.5%), Benzoic acid, 3,4-dihydroxy (7.4%), Tyrosol (4.8%), Diacetone alcohol (4.0%), 7,9-Di-tert-butyl-1-oxaspiro[4.5]deca-6,9-diene-2,8-dione (3.6%), 1,2,3,4-Tetramethyl-3-benzene (3.4%), dihydro-sinapyl alcohol (3.2%), m-Pentadecylpheno (2.4%), Fern-9(11)-en-28-ol (2.3%), and Phenol, 3-undecyl (2.2%) emerged as the most prevalent compounds in the PBM culture medium.

### Effect of cultivation time

**Fig. 1:**
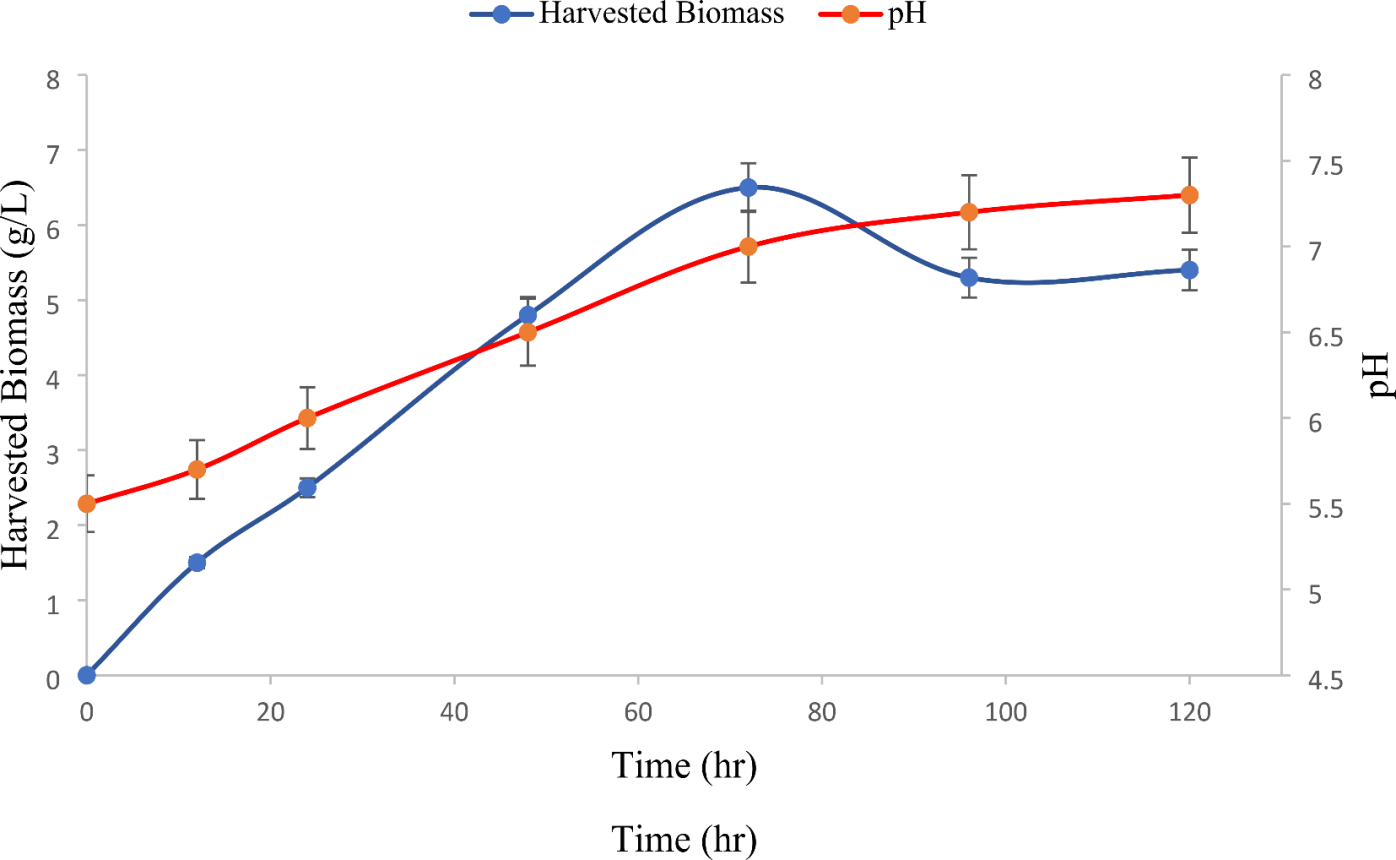
Profile of the of harvested biomass and pH during cultivation of N.intermedia in PBM culture media at initial pH of 5.5 and temperature of 30 ^0^C.

The impact of cultivation duration on biomass quantity, pH, and protein content was examined (Fig. 1). A 72-hour cultivation period yielded the highest fungal biomass concentration of 6.7 g/L with a protein content of 20.4% on a dry weight basis or on the other hand, 26.8 g of dried fungal biomass per kilogram of PBM (Given the high water absorption capacity of PBM, the culture medium obtained after pretreatment was 40% of the initial pretreatment solution. The calculated yield was reported based on this reduced volume). Deviations from this optimal cultivation time, either shorter or longer, resulted in significantly reduced biomass levels. Upon closer investigation, the lag phase for *N. intermedia* growth in this medium was approximately 8 h, aligning with previous studies on ethanol and biomass production from thin stillage utilizing *N. intermedia*^23^. The maximum biomass productivity of 125 mg·L^− 1^·h^− 1^ was attained following a 15-hour cultivation period. Throughout a 120-hour timespan, the pH value underwent a transition from 5.5 to 7.3. At the 72-hour mark of the cultivation process, a pH value of 7 was ascertained as the optimal time frame corresponding to the highest biomass quantity. An elevation in pH levels is a common occurrence in fungal cultivations that involve protein hydrolysis or biochemical such as ethanol production^24^. Various other research works have corroborated the necessity of a 72-hour cultivation duration to achieve the maximum biomass yield^25,26^. Within the context of this investigation, the fungal biomass yield in grams per gram of initial total COD stood at 0.37 g/g. Moreover, the findings derived from examining the impact of cultivation duration and resulting yield align with a previous study utilizing fish industry by-products for cultivating *Rhizopus oryzae*, wherein the fungal biomass / total COD (g/g) yield ranges from approximately 0.25 to 0.65 after 72 h based on the specific substrate utilized^27^. Also, this yield is consistent with the results of some studies regarding the treatment of industrial wastewater with filamentous fungi^28^.

**Fig. 2:**
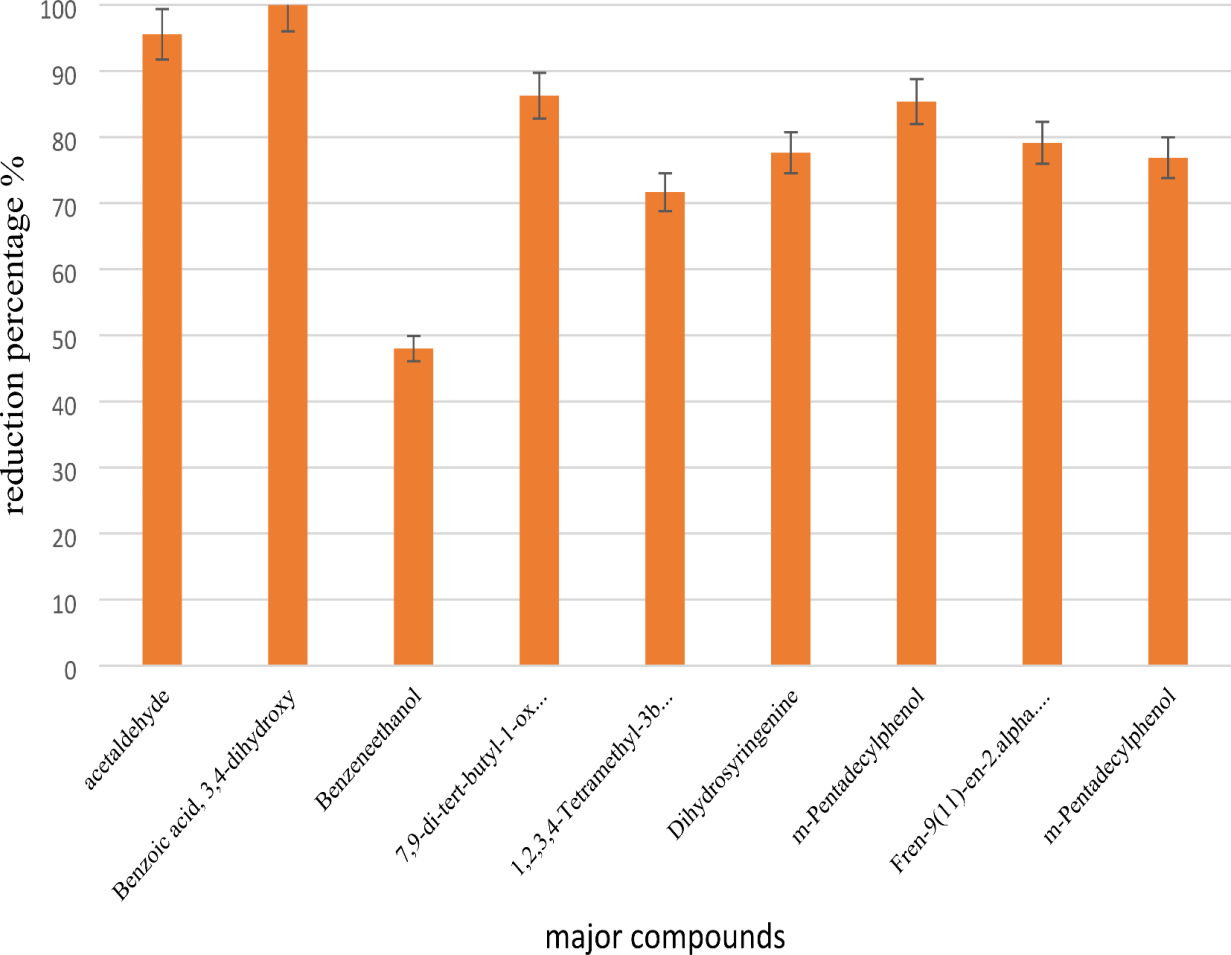
Percentage reduction of major compounds identified by GC-MS analysis after N.intermedia cultivation.

Following the harvest of fungal biomass, an analysis using GC-MS was conducted on the residual culture medium, leading to the identification of 64 compounds, with 45 being consistent with those found in the primary culture medium. The remaining compounds were detected in minimal quantities that were deemed insignificant. The reduction percentage of the key compounds identified in the primary culture medium is depicted in Fig. 2.

These compounds predominantly exhibit phenolic and aromatic characteristics. Owing to the broad enzymatic capabilities possessed by filamentous fungi, they can adapt to adverse conditions, including the presence of highly hazardous substances such as phenolic, aromatic, and alcoholic compounds. Filamentous fungi demonstrate proficiency in degrading a variety of aromatic compounds^29^. For example, it was reported that *aspergillus awamori* strain was capable of degrading and utilizing as single carbon source 1 g/L phenol, 3 g/L catechol, 2 g/L dichlorophenol, 1 g/L 2,6-dimethoxyphenol within 6–7 days of its development^30^. It is well known that phenolic compounds are metabolized by a 3-oxoadipate pathway and enters central metabolism as succinate and acetyl-S-CoA^31,32^. Despite the ambiguity surrounding the degradation mechanism of filamentous fungi in their natural habitat, they are equipped with enzymes that facilitate biotransformation, involving oxidation and hydroxylation reactions. Enzymes like peroxidase, monooxygenases, laccases, hydroxylase, and hydrolase possess the capacity to oxidize a diverse array of substrates, transferring electrons from organic compounds to molecular oxygen through oxidation-reduction reactions or epoxidation. Additionally, intracellular enzymatic systems, internal mechanisms, and Fenton reactions represent other degradation pathways that collectively underscore the adaptability and versatility of filamentous fungi^33^.

### Effect of initial pH

**Fig. 3:**
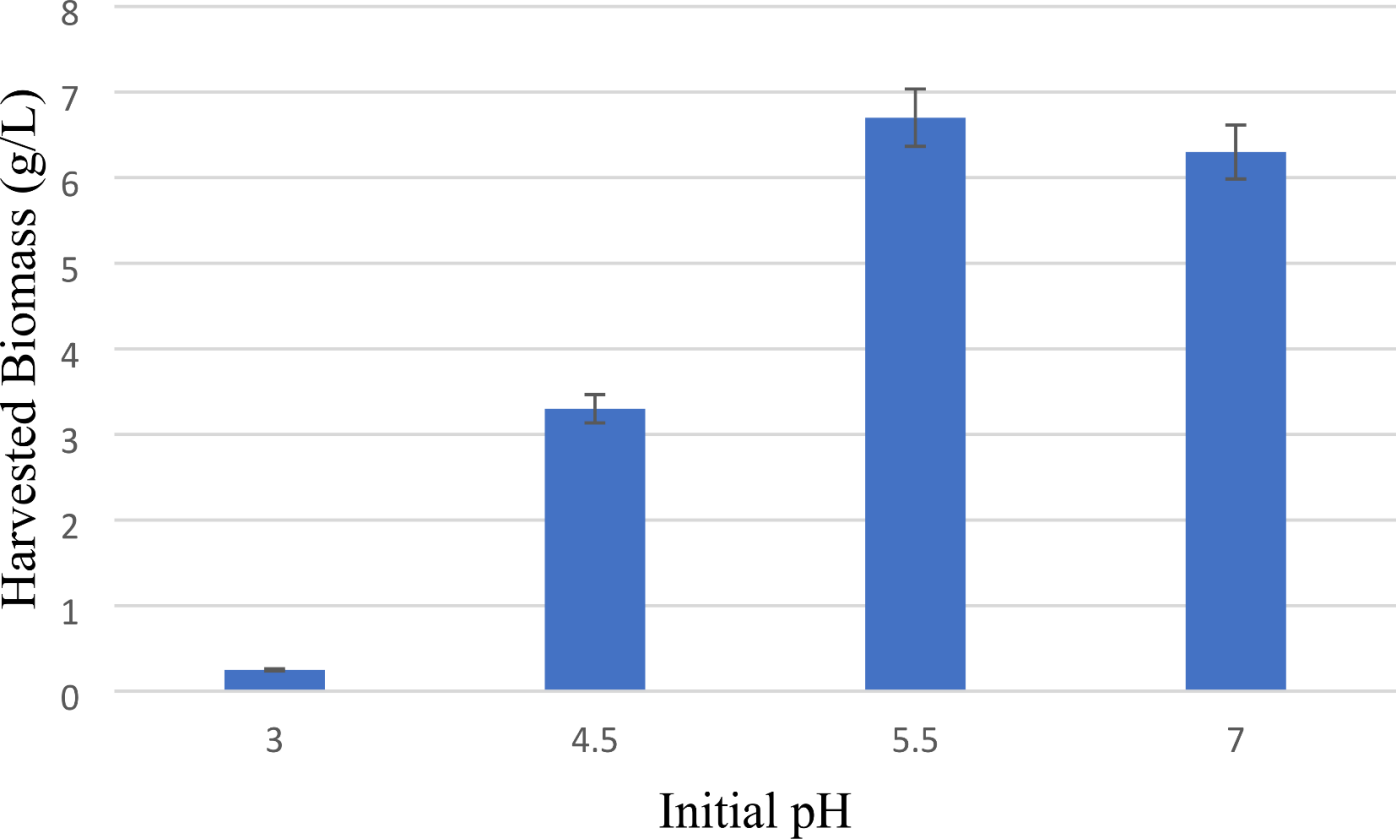
Effect of initial pH on harvested N. intermedia after 72 h and temperature of 30 ^0^C.

The investigation conducted in this research demonstrated that the initial pH had a notable impact on the development of fungi, as illustrated in Fig. 3. A higher initial pH of 5.5 resulted in the most substantial biomass production of 6.7 g/L after 72 h, accompanied by a protein content of 20.4 ± 0.5%, equivalent to the protein content under optimal cultivation conditions. Conversely, a decrease in the initial pH levels (3 and 4.5) led to a considerable reduction in fungal biomass. Limited growth was observed at pH 4.5, while negligible growth occurred at pH 3, primarily attributed to the minimal microbial activity in acidic conditions (below pH 5)^34^. In contrast to the conditions at pH 5.5, characterized by filamentous mycelial clump growth, a greater prevalence of pellet growth was evident at pH 4.5. The pH range of 3.0–4.0 is recognized as a highly influential factor for the formation of pellets in *N. intermedia* cultures^35^. It was noted that the pellet cultures developed at pH 4.5 exhibited a higher ethanol yield compared to the filamentous mycelial clump form, with values of 0.35 g ethanol/ initial total COD at pH 4.5 and 0.29 g ethanol/ initial total COD at pH 5.5 after 72 h. This result is in consistent with Nair, et al.^35^ investigation of mycelial pellet formation by *N. intermedia*.

The optimal pH value determined for the cultivation of *N. intermedia* in this study (pH 5.5) aligns with findings from previous research, particularly studies involving the use of vinasse as an alternative nutrient source for fish feed in *N. intermedia* cultivation^25^. Exploring the industrial-scale production of fungal biomass from PBM extract involving *N. intermedia* underscores the significance of maintaining the original medium pH as the optimal cultivation pH.

To assess the influence of a constant pH on fungal biomass generation, the medium’s pH was regulated to the optimal initial value of 5.5 throughout the cultivation period. Subsequently, after 72 h of cultivation, a dry fungal biomass yield of 8.8 g/L with a protein content of 18.6% was achieved, corresponding to a biomass yield of 0.49 g/g of initial Total COD. Maintaining a stable pH level is known to enhance *N. intermedia* fungal biomass production significantly compared to uncontrolled pH conditions due to enhanced enzyme activity and improved nutrient uptake under slightly acidic pH conditions.

### Effect of temperature

**Fig. 4:**
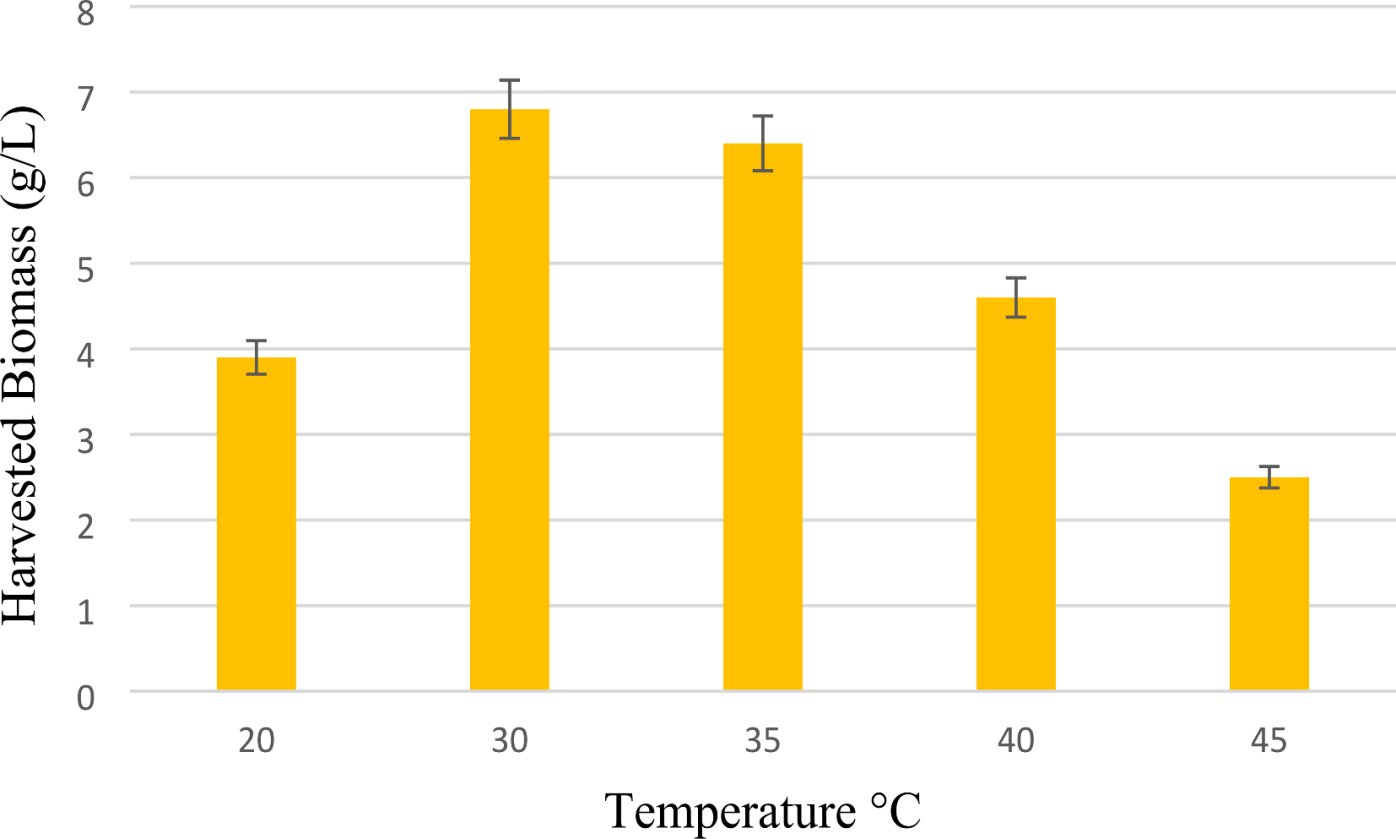
Effect of Temperature on harvested N.intermedia after 72 h and pH 5.5.

Given that *N. intermedia* is a mesophilic strain, the temperature range of 20–45 °C was explored at an initial pH of 5.5 over a 72-hour period. The data presented in Fig. 4 indicated that the most favorable biomass growth of 6.7 g/L was attained at 30 °C, consistent with previous findings. In light of optimizing biomass growth and energy efficiency, a temperature of 30 °C is considered the most conducive growth condition. Therefore, meticulous temperature regulation is imperative for industrial-scale applications to ensure optimal growth, energy conservation, and mitigate heat stress-induced damage to the fungus. The obtained result is consistent with various other studies. For example, Ferreira, et al.^23^ investigated the different temperature of 25–45 °C to produce ethanol and biomass from thin stillage by using various Zygomycetes and Ascomycetes Filamentous Fungi. In this study the optimal temperature to obtain maximum amount of *N. intermedia* biomass was considered 30 °C. In another study, Hashemi et al.^36^ produced the protein-rich biomass of *N. intermedia* from baker’s yeast wastewater. The biomass yield in the temperature of 30 °C and pH 5.3 was obtained 2.75 g DM/L.

The impact of temperature on the growth of *N. intermedia* biomass is intricate and influenced by various factors, such as enzyme activity and nutrient absorption^37–39^. The deceleration in the growth rate of *N. intermedia* occurs at temperatures approximately 20 °C, possibly due to the decreased efficiency of enzyme reactions and cellular processes that propel growth at lower temperatures. Moreover, nutrient absorption may be hindered at colder temperatures owing to reduced membrane fluidity and diminished enzymatic activity. Elevated temperatures can also impede cellular function and biomass growth by damaging cellular proteins, lipids, DNA, denaturing proteins, and compromising membrane integrity^28,37–39^. The upper limit for the growth of mesophilic fungi is approximately 48 °C^40^.

### Effect of inoculation methods

The enhancement in fungal biomass growth can be achieved through the presence of various phenolic and aromatic compounds in the PBM extract medium, as well as acclimatization of the inoculum and adaptation of *N. intermedia*. In order to explore the impact of inoculum acclimatization and *N. intermedia* adaptation on fungal biomass growth, the primary PBM culture medium was diluted twice. A spore suspension of 2.5% v/v was introduced to the diluted medium, followed by fermentation at 30 °C for 24 h with an initial pH of 5.5. Subsequently, 10% v/v of a solution containing pre-cultured mycelial clump was added to the primary PBM culture medium. After 72 h, a dry fungal biomass of 7.9 g/L with a protein content of 20.2% was attained, resulting in a yield of 0.44 g/g of fungal biomass per gram of initial Total COD. Although the protein content did not significantly differ from the optimal fungal biomass protein content (20.4%), the biomass production increased by 18%. The heightened fungal biomass production through the pre-cultured inoculum method can be attributed to a more uniform and concentrated inoculum, faster germination, and improved nutrient uptake compared to a spore suspension. The adaptation of *N. intermedia* to the cultivation medium aligns with the findings of previous studies on inoculation methods^41^. For instance, in the investigation conducted by Nair, et al.^35^ on the formation of mycelial pellets by *N. intermedia*, altering the inoculation method resulted in varying fungal biomass yields. Under identical operating conditions (pH of 3 and cultivation time of 96 h), biomass yields of 0.115, 0.122, and 0.195 g/g of consumed glucose were obtained using pellet, spore, or mycelial clump inoculum, respectively.

### Effect of adding nitrogen source

**Fig. 5:**
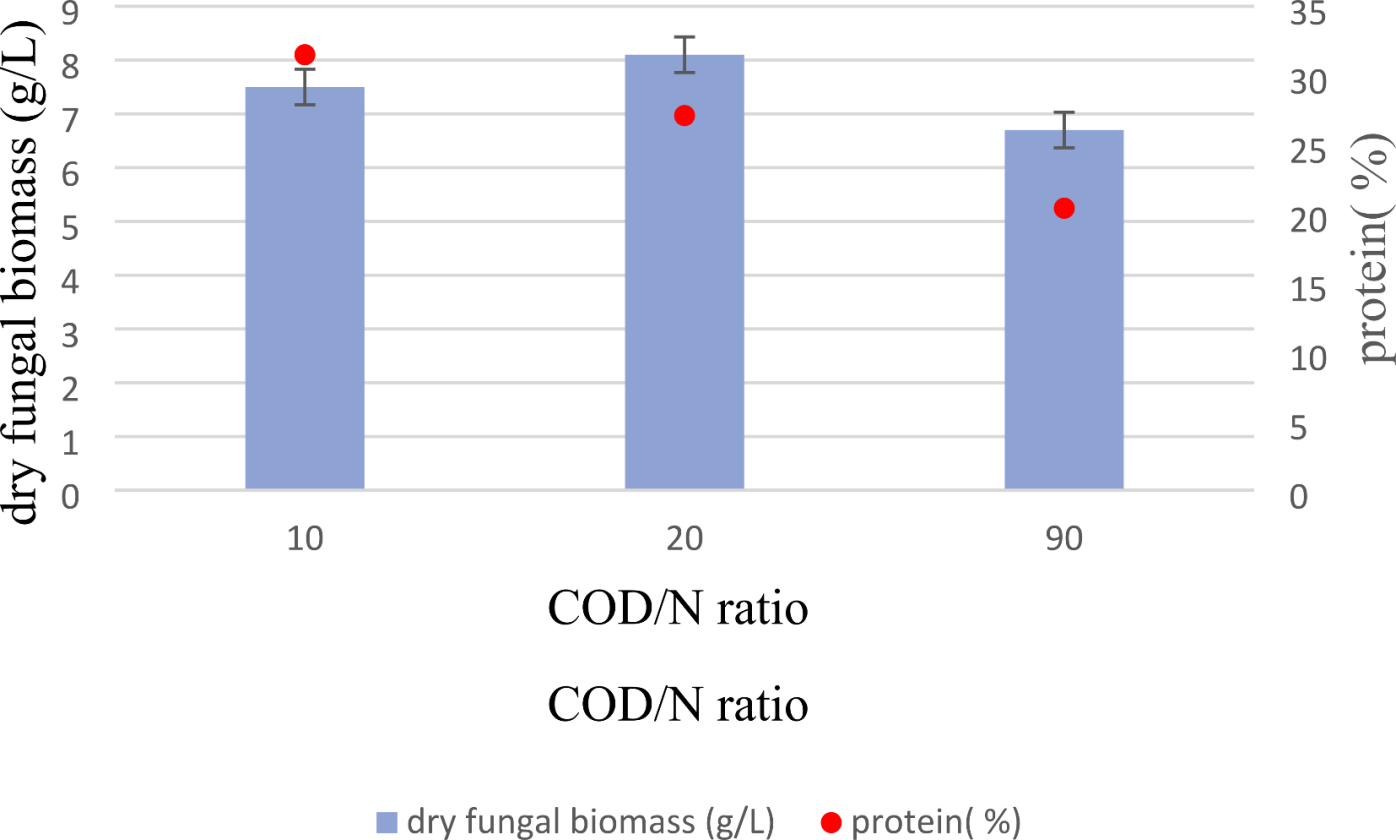
Effect of adding nitrogen source on harvested N.intermedia after 72 h, pH 5.5 and temperature of 30 ^0^C.

The optimal operational conditions, specifically a cultivation period of 72 h, temperatures of 30 °C, and an initial pH of 5.5, resulted in the attainment of 6.7 g/L of dry fungal biomass in the PBM medium without nitrogen supplementation, maintaining a COD/N ratio of 90. Within the nitrogen-deficient PBM medium, the introduction of nitrogen sources led to an enhancement in both biomass and protein production efficiency. As the data presented in Fig. 5, Manipulating the COD/N ratio to 20 and 10 resulted in an increase in dry fungal biomass to 8.1 and 7.5 g/L, respectively. The promotion of *N. intermedia* biomass growth was observed when supplementing the media with NaNO_3_ as an inorganic nitrogen source, reflecting the preference of most fungi for inorganic nitrogen over organic nitrogen^28^. The outcomes of this study highlight the significant influence of the COD/N ratio on fungal biomass production and protein content. Protein levels reached 27.1% and 31.4% for COD/N ratios of 20 and 10, respectively, indicating a substantial increase of approximately 33% and 54% in fungal biomass protein content. With response of fungal biomass growth, COD/N ratio of 20 showed higher yield of 0.45 g of fungal biomass per gram of initial total COD.

Lower COD/N ratios are known to result in biomass with higher protein content^28^. While the addition of nitrogen sources to the medium may incur additional processing expenses in industrial settings, it yields high-value products, such as increased protein content in fungal biomass, suitable for nonruminant consumption^42^. Furthermore, the incorporation of protein- or nitrogen-enriched waste or wastewater, such as fish processing wastewaters, into PBM can serve as a nitrogen source, warranting further investigation. This avenue of research could lead to novel approaches for bioconversion of PBM and wastewater treatment utilizing edible filamentous fungi. Numerous research endeavors have explored the nitrogen sourcing in culture media. Hashemi, et al.^43^ conducted a study on *N. intermedia* cultivation using vinasse and whey for protein and biogas production. The addition of extra nitrogen source to the culture medium (vinasse to whey ratio of 25:75, v/v) increased fungal biomass yield from 10.2 to 12.0 g/L after 96 h. This observation aligns with the results of the current study, indicating an upward trend in fungal biomass and protein content production through nitrogen supplementation.

## Conclusion

Utilizing *N. intermedia* as a robust filamentous fungus Along with hot water extraction pretreatment method presents a promising strategy for generating protein-rich fungal biomass as value-added products from PBM, an agro-industrial waste characterized by high COD levels and low sugar content, unlike conventional culture media rich in sugars. The substantial increase in fungal biomass quantity and protein content achieved through pre-cultured inoculation, nitrogen supplementation, and pH regulation during cultivation could reignite research interest in employing filamentous fungi for PBM management. The highest fungal biomass yield was 0.49 g of fungal biomass per gram of initial total COD and for protein content was 27.1%. The findings of this research, by creating a comprehensive value chain and transforming PBM into high-value products which mentioned in our previous study^2^, have taken a significant step towards establishing a sustainable and promising bioeconomy. This achievement will not only strengthen the regional economy but also contribute significantly to environmental.

## Data Availability

This published paper includes all the data produced or analyzed during the study.
